# The association between national culture and AI readiness: a cross-national study

**DOI:** 10.3389/frai.2026.1727606

**Published:** 2026-02-11

**Authors:** Kumiko Komatsu, Nina Ždanovič, Masaki Yamabe, Hiroyoshi Iwata, Misa Iwamoto, Shutaro Takeda

**Affiliations:** Graduate School of Media Design, Keio University, Yokohama, Japan

**Keywords:** artificial intelligence, cross-cultural studies, Hofstede’s cultural dimensions, national culture, technology adoption

## Abstract

While the adoption of Artificial Intelligence (AI) is advancing globally, its pace varies significantly across nations. This study statistically examines the associations between Hofstede’s cultural dimensions and national-level AI readiness. A correlation analysis was conducted using data from the Oxford Insights’ “Government AI Readiness Index 2024” and Hofstede’s cultural dimension scores. The findings reveal that Individualism and Long-Term Orientation have a significant positive correlation with AI readiness, whereas Power Distance and Uncertainty Avoidance show a significant negative correlation. Conversely, Masculinity and Indulgence did not have a statistically significant relationship. These results suggest that national cultural characteristics are associated with differences in the adoption of advanced technologies such as AI. To contextualize the statistics, we include an illustrative, non-causal comparison of Japan, the United States, and Singapore.

## 1 Introduction

Artificial Intelligence (AI) has emerged as a transformative force with the potential to fundamentally reshape the global economy by adding trillions of dollars in value and significantly boosting productivity (Chui, 2023). The ramifications of the global AI skills gap extend far beyond purely economic concerns, creating ripple effects that permeate societal structures and threaten to reshape the global order (Wu and Lin, 2024). Similarly, the diffusion of generative AI technologies may exacerbate inequalities across domains such as education, work, and healthcare, demanding comprehensive policy interventions (Capraro et al., 2024). More broadly, cross-national research on innovation and diffusion highlights systematic differences in how quickly new technologies spread across countries. Prior work also suggests that national cultural orientations can be associated with cross-country variation in innovation rates and diffusion dynamics (Jaiswal and Zane, 2022). Against this backdrop, governments worldwide are increasingly integrating AI into public services, making AI readiness an important national capability (Oxford Insights, 2024). Research on technology acceptance has highlighted the role of users’ beliefs in shaping adoption, as exemplified by the Technology Acceptance Model (TAM). In TAM, these beliefs primarily refer to perceived usefulness and perceived ease of use, which influence users’ attitudes and intention to use (Davis, 1989). However, to explain disparities in AI adoption at the national level, a macro-level perspective that considers cultural dimensions is critical, beyond just economic and policy factors.

Several prior studies have examined the relationship between cultural values and technology adoption across nations. For example, differences in information and communication technologies (ICT) uptake can be partially explained by cultural dimensions such as uncertainty avoidance and individualism (Erumban and de Jong, 2006). Likewise, national culture has been shown to influence both managerial perceptions and organizational use of IT (Leidner and Kayworth, 2006). Taken together, this literature suggests that national culture may shape technology uptake, for example through differences in risk-related orientations and authority relations that can influence managerial perceptions and organizational use of IT. Building on this literature, we extend the inquiry to the national AI readiness context by examining statistical associations between Hofstede’s cultural dimensions and the Government AI Readiness Index (Oxford Insights, 2024).

Hofstede’s seminal argument that organizational practices and theories are culturally relative (Hofstede, 1983) provides critical insight for understanding how national cultural values relate to country-level technology adoption and AI readiness. Building on this argument, our study argues that a nation’s collective stance toward a transformative technology like AI is similarly shaped by its deep-seated cultural values. The purpose of this paper is to systematically investigate this underexplored relationship.

Building on the above, we anchor our analysis in two theoretical pillars: national AI readiness and Hofstede’s model of cultural dimensions. For national AI readiness, we adopt the Government AI Readiness Index from Oxford Insights, which conceptualises AI readiness as governments’ readiness to implement AI in the delivery of public services and operationalises it using 40 indicators across three pillars—Government, Technology Sector, and Data & Infrastructure (Oxford Insights, 2024). We interpret this composite score as reflecting governments’ overall preparedness and capacity for public-sector AI implementation, while noting that it also includes some adoption-related indicators.

On the cultural side, we draw on Hofstede’s six-dimensional model of national culture (Hofstede, 2025a,b). While the model has limitations—such as reliance on historical data from a single multinational company (IBM) and its static treatment of culture—it remains a widely used framework for quantitative cross-cultural research. Consistent with this, a recent meta-analysis of technology acceptance studies documents the continued incorporation of Hofstede’s dimensions across diverse adoption contexts (Jan et al., 2024).

At the country level, prior research suggests that national culture is associated with patterns of technology adoption and diffusion, and that these relationships are often studied at macro and organizational levels rather than being reducible to individual acceptance alone. Drawing on reviews of culture in IS research highlighting how cultural values shape IT management and implementation dynamics (Leidner and Kayworth, 2006), and evidence that cross-country differences in ICT adoption can be examined using Hofstede’s cultural dimensions (Erumban and de Jong, 2006), we treat Hofstede’s dimensions as macro-level orientations potentially relevant to coordination, risk handling, and long-term capability building. Relatedly, cross-national diffusion research links national culture and attitudes to differences in how new technologies spread across countries (Jaiswal and Zane, 2022).

Hypothesis development. Building on cross-national evidence that Hofstede’s national culture dimensions are statistically associated with country-level innovation indicators (Espig et al., 2022) and evidence that Hofstede’s dimensions are widely applied in technology adoption studies (Jan et al., 2024), we derive directional expectations for each dimension. We expect Individualism (IDV) to be positively associated with AI readiness, as cultures emphasizing autonomy and individual initiative tend to reward innovative effort and experimentation, and prior country-level evidence links individualism to both innovation outcomes and the uptake of new technologies (Erumban and de Jong, 2006; Gorodnichenko and Roland, 2011). We expect Long-Term Orientation (LTO) to be positively associated with AI readiness because future-oriented cultures tend to emphasize perseverance and long-term planning, often channeling effort into education and economic modernization, and may also shape technology acceptance by making long-run benefits more salient during technology rollouts (Espig et al., 2022; Jan et al., 2024). Power Distance (PDI) is expected to be negatively associated with AI readiness, as high-PDI contexts tend to feature centralized decision structures and hierarchical authority relations that can restrict communication and information sharing, potentially complicating broad implementation efforts (Hofstede, 1983; Espig et al., 2022). Uncertainty Avoidance (UAI) is expected to be negatively associated with AI readiness because high-UAI societies tend to avoid uncertain or unproven options, prefer stability and established routines, and are less willing to take risks or experiment with disruptive technologies (Erumban and de Jong, 2006; Espig et al., 2022). We also expect Masculinity (MAS) to be negatively associated with AI readiness insofar as achievement- and competition-oriented cultures may place less emphasis on cooperation, consensus, and error tolerance, which can undermine the collaborative and learning-oriented processes required for responsible public-sector AI implementation (Hofstede, 1983; Espig et al., 2022). Finally, Indulgence (IVR) is expected to be negatively associated with AI readiness, as more indulgent cultures may exhibit lower acceptance of—and compliance with—stringent, coordination-intensive public interventions, thereby weakening large-scale implementation capacity. This expectation is consistent with cross-national evidence that the effectiveness of restrictive government measures is attenuated in more indulgent contexts (e.g., compliance with COVID-19 control policies) (Xu et al., 2024; Lee et al., 2025).

Therefore, the central research question of this paper is: “How are a nation’s cultural characteristics, as defined by Hofstede, statistically associated with its AI readiness?” To answer this question, this study performs a cross-national quantitative analysis, correlating Hofstede’s cultural dimensions with the Government AI Readiness Index (Oxford Insights, 2024).

While national AI readiness is often discussed in terms of structural economic conditions and policy capacity, a complementary perspective is to examine whether it is statistically associated with macro-level cultural orientations. This study makes three contributions. First, we examine the statistical associations between Hofstede’s six cultural dimensions and the Government AI Readiness Index using publicly available country-level data. Second, we report Pearson correlation analyses and a multiple linear regression model (OLS) to compare bivariate associations with estimates from a model including all six dimensions simultaneously. Third, we provide a brief, illustrative (non-causal) comparison of Japan, the United States, and Singapore to contextualize the statistical patterns.

## 2 Methods

This study employs a quantitative, cross-national research design to investigate the relationship between national cultural dimensions and AI readiness. The methodology is structured into two parts—data collection and data analysis—and includes a brief, non-causal illustrative comparison of three high-readiness countries (Japan, the United States, and Singapore) to contextualize the results.

### 2.1 Data collection and sample

The dataset for this study was compiled from two distinct, publicly available sources.

#### 2.1.1 Dependent variable

AI readiness: the dependent variable, national AI readiness, was measured using the overall score (on a scale of 0 to 100) from the “Government AI Readiness Index 2024” (Oxford Insights, 2024). This index provides a comprehensive assessment of a country’s preparedness for AI, and specifically, it is a composite measure designed to assess how ready governments are to implement AI in the delivery of public services. It is built from 40 indicators organized into three pillars (Government, Technology Sector, and Data & Infrastructure) and aggregated into an overall score that is normalized on a 0–100 scale, where higher values indicate greater national readiness for public-sector AI adoption and implementation. In this study, the overall index score was treated as a continuous dependent variable in the analysis.

#### 2.1.2 Independent variables

Cultural dimensions: the independent variables were the six cultural dimension scores for each country, as defined by Hofstede. The data were retrieved from the “Dimension data matrix” (version 2015-12-08) published on Geert Hofstede’s website (Hofstede, 2025a,b). We used the modified 0–100 dataset provided on the same page. Hofstede’s national cultural dimensions were operationalized using country scores for the six-dimension model (PDI, IDV, MAS, UAI, LTO, IVR) from the Hofstede dimension data matrix. Each dimension is reported as a country-level score on a 0–100 scale, where higher values indicate a stronger presence of the named cultural tendency. Specifically, Power Distance (PDI) reflects acceptance of unequal power distribution; Individualism (IDV) reflects preference for individual autonomy over group cohesion; Masculinity (MAS) reflects emphasis on competition and achievement over cooperation and quality of life; Uncertainty Avoidance (UAI) reflects discomfort with ambiguity and preference for rules and predictability; Long-Term Orientation (LTO) reflects future-oriented pragmatism and perseverance; and Indulgence (IVR) reflects a greater tendency toward gratification and enjoyment rather than restraint. These scores were treated as continuous independent variables in the analysis (Hofstede, 2025a,b).

The final sample consists of 61 countries with available data in both the Government AI Readiness Index and Hofstede’s cultural dimension dataset. Countries were excluded if either the AI readiness score or any of the six Hofstede scores was missing in the source datasets. We used complete-case analysis (i.e., no imputation), retaining only countries with complete data across both sources. The resulting sample spans multiple world regions; however, regional coverage may be uneven due to differences in data availability across the two sources.

### 2.2 Data analysis

The statistical analysis was conducted using Python programming language. Key libraries included ‘pandas’ for data handling, ‘scipy.stats’ for Pearson correlation analysis, and ‘statsmodels’ for multiple linear regression modeling.

To investigate the relationships between Hofstede’s six cultural dimensions and the national AI readiness score, two main methods were employed. First, Pearson correlation analysis was used to assess the linear association between each cultural dimension and AI readiness. Second, a multiple linear regression analysis was conducted to estimate the relative contribution of each cultural dimension in predicting AI readiness. A *p*-value of less than 0.05 was considered statistically significant for all analyses.

### 2.3 Screening and assumption checks

Screening and assumption checks. We conducted screening procedures prior to inference. Countries were retained only when the AI readiness score and all six Hofstede dimension scores were available (complete-case analysis; no imputation). Potential outliers were screened using standardized *z*-scores across the dependent variable and the six predictors (threshold |*z*| > 3), and no countries exceeded this threshold. Regression assumptions were assessed on the OLS model residuals. Normality was evaluated using Q–Q plots of residuals, and linearity/homoscedasticity were inspected via residuals-versus-fitted plots. We additionally conducted the Breusch–Pagan test for heteroscedasticity, which was not statistically significant. Multicollinearity was diagnosed using variance inflation factors (VIF) and tolerance values; VIFs were low (all < ~2.1) and tolerances were acceptable (all > ~0.49), indicating no severe multicollinearity among predictors. Overall, diagnostics did not suggest major violations of regression assumptions; however, given the cross-sectional design and the use of secondary indicators, findings should be interpreted cautiously as associations rather than causal effects.

## 3 Results

This section presents the statistical findings of the study. First, the descriptive statistics for all variables used in the analysis are summarized. Then, the results of the Pearson correlation analysis are reported, followed by the multiple regression analysis to examine the simultaneous effects of all cultural dimensions on AI readiness.

### 3.1 Descriptive statistics

The descriptive statistics for the 61 countries included in this study are presented in Table 1. This table provides the number of observations (*N*), mean, standard deviation, and the minimum and maximum values for the AI Readiness Index score and each of the six Hofstede’s cultural dimensions (see Table 1 for details).

**Table 1:** Descriptive statistics and Pearson correlations with AI readiness.

#	Variable	*N*	Mean	Std. Dev.	Min	Max	Correlation coefficient (*r*)	*p*-value
1	Individualism (IDV)	61	46.1	23.72	12	91	0.55	<0.001***
2	Long-Term Orientation (LTO)	61	49.57	23.01	13	100	0.47	<0.001***
3	Power Distance (PDI)	61	58.33	20.49	11	100	−0.38	0.002**
4	Uncertainty Avoidance (UAI)	61	67.26	22.28	8	100	−0.31	0.015*
5	Masculinity (MAS)	61	48.82	19.96	5	100	−0.097	0.459
6	Indulgence (IVR)	61	48	22.2	0	100	−0.012	0.924

Significance codes: *p < 0.05, **p < 0.01, ***p < 0.001.

Interpretation of Descriptive Statistics: Table 1 demonstrates considerable variation across countries. The AI readiness scores span a wide range (29.21–87.03), and cultural dimensions such as IDV and UAI also show substantial diversity.

### 3.2 Correlation analysis

**Figure 1:**
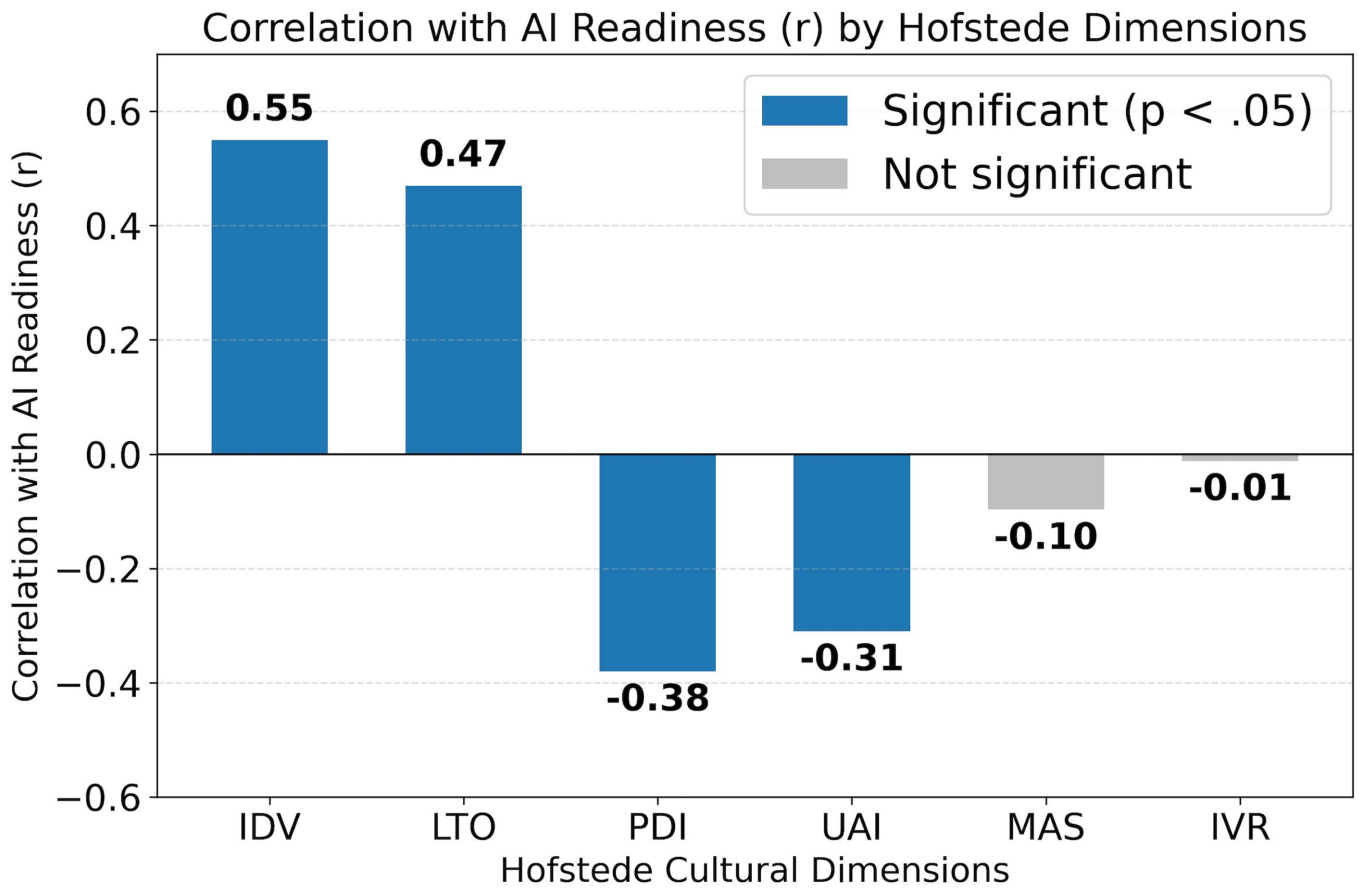
Bar chart showing the Pearson correlation between Hofstede’s cultural dimensions and the AI readiness index (*N* = 61).

To assess the direct relationship between each cultural dimension and AI readiness, a Pearson correlation analysis was conducted (see Figure 1). The results, as detailed in Table 1, indicate that four of the six dimensions have a statistically significant correlation with the AI readiness score (see Table 1 for details).

AI readiness was positively associated with Individualism (IDV) (*r* = 0.55, *p* < 0.001) and Long-Term Orientation (LTO) (*r* = 0.47, *p* < 0.001), and negatively associated with Power Distance (PDI) (*r* = −0.38, *p* = 0.002) and Uncertainty Avoidance (UAI) (*r* = −0.31, *p* = 0.015). Masculinity (MAS) and Indulgence (IVR) were not significant (see Figure 1).

### 3.3 Multiple regression analysis

To investigate which cultural dimensions predict AI readiness while controlling for the other dimensions, we estimated an OLS multiple regression model (see Table 2 for details).

**Table 2:** Multiple regression analysis results.

Variable	Coefficient (*B*)	Std. Error	Standardized coefficient (*β*)	*p*-value
(Constant)	46.81	9.37	—	<0.001***
Power Distance (PDI)	0.003	0.077	0.00	0.972
Individualism (IDV)	0.224	0.064	0.44	0.001**
Masculinity (MAS)	−0.078	0.058	−0.13	0.184
Uncertainty Avoidance (UAI)	−0.099	0.052	−0.18	0.061
Long-Term Orientation (LTO)	0.271	0.057	0.52	<0.001***
Indulgence (IVR)	0.099	0.063	0.18	0.121

Significance codes: *p < 0.05, **p < 0.01, ***p < 0.001.

The overall model was statistically significant, *F*(6, 54) = 10.83, *p* < 0.001, with *R*^2^ = 0.546 (adjusted *R*^2^ = 0.496; *N* = 61). Individualism and Long-Term Orientation emerged as the only statistically significant predictors. As effect-size indicators, standardized coefficients showed the largest positive associations for Individualism (*β* = 0.44) and Long-Term Orientation (*β* = 0.52).

The discrepancy between the Pearson correlation results and the OLS multiple regression results is not necessarily contradictory. Pearson correlations reflect the bivariate associations between each cultural dimension and AI readiness, whereas the regression coefficients quantify each dimension’s unique association with AI readiness while controlling for the other Hofstede dimensions. Because Hofstede dimensions are intercorrelated, shared variance across dimensions can lead some predictors to lose statistical significance once all six dimensions are modeled simultaneously, even when their Pearson correlations are significant. In this study, multicollinearity diagnostics (VIF/tolerance; see Section 2.3) did not indicate severe multicollinearity; however, the shared variance among cultural dimensions implies that the regression results should be interpreted as conditional estimates within the joint model, rather than as definitive conclusions about “the most important” dimension. Thus, we describe IDV and LTO as the dimensions that remained significantly associated with AI readiness in the joint model for the current sample.

## 4 Discussion

### 4.1 Cultural dimensions and their associations with AI readiness

This study initially hypothesized that all six of Hofstede’s cultural dimensions would be associated with national AI readiness. However, the results reveal that only four dimensions—Individualism (IDV), Power Distance (PDI), Uncertainty Avoidance (UAI), and Long-Term Orientation (LTO)—demonstrated statistically significant relationships. In contrast, Masculinity (MAS) and Indulgence (IVR) did not show statistically significant associations, suggesting that not all cultural traits are equally associated with a country’s preparedness for AI.

First, individualism (IDV) showed the strongest positive association. Prior cross-country work suggests that more individualistic societies tend to be more receptive to innovations and new ideas, which can contribute to higher uptake of new ICT (Erumban and de Jong, 2006). This suggests that higher levels of individualism are positively correlated with greater AI readiness.

Second, Power Distance (PDI) was negatively associated with AI readiness. In high-PDI cultures, hierarchical decision-making may be associated with less open information flow, which may correspond to lower national AI readiness. This aligns with evidence that higher power distance can restrict information sharing and thereby hinder the introduction of new products and innovations (Espig et al., 2022). Therefore, higher power distance is negatively correlated with AI readiness.

Third, Uncertainty Avoidance (UAI) also showed a significant negative correlation with AI readiness. Cultures with low tolerance for ambiguity may be associated with slower adoption of emerging technologies when safety, regulation, or precedent is perceived as insufficient. This aligns with broader research showing that high UAI is associated with reduced openness to innovation (Espig et al., 2022), and that in high-UAI societies, even potentially innovative changes—such as remote work setups—are less likely to foster innovation (Wang et al., 2025). Therefore, higher uncertainty avoidance is associated with lower AI readiness.

Fourth, Long-Term Orientation (LTO) was positively related to AI readiness. Consistent with this, recent evidence reports that LTO is associated with citizens’ behavioral intention toward technology adoption (Guo, 2024), and meta-analytic results suggest that LTO positively moderates key correlates of behavioral intention in technology acceptance (Jan et al., 2024). These societies prioritize future rewards over short-term gains, aligning with the long-term benefits associated with AI systems. In other words, stronger Long-Term Orientation is positively correlated with higher AI readiness.

By contrast, masculinity (MAS) and indulgence (IVR) did not demonstrate statistically significant associations. These findings suggest that competitiveness (as reflected in masculinity) and pursuit of pleasure or gratification (as reflected in indulgence) are not key correlates of AI readiness at the national level. Thus, neither greater masculinity nor indulgence appears to promote or hinder AI readiness in a meaningful way.

Taken together, the pattern across the four significant dimensions suggests a coherent direction. This concerns national cultural characteristics—particularly those that value individual autonomy (higher IDV) and future-oriented planning (stronger LTO). These cultural dimensions may function as important enablers of the adoption of advanced technologies such as AI; in contrast, higher hierarchy (PDI) and higher uncertainty avoidance (UAI) may act as cultural frictions that are associated with slower diffusion. These findings directly answer the central research question by showing that Hofstede’s cultural dimensions are statistically associated with national AI readiness—positively for IDV and LTO, and negatively for PDI and UAI. Understanding how these values are statistically associated with higher or lower AI readiness provides important insights for designing national AI strategies that are socially and culturally aligned.

### 4.2 Cross-national comparison: Japan, the United States, and Singapore

This subsection examines three countries—the United States, Japan, and Singapore—as illustrative cases to contextualize the cross-national results. These cases were selected because (i) all three score highly on the Government AI Readiness Index, providing a common baseline of high national AI readiness, and (ii) they exhibit contrasting cultural profiles on key Hofstede dimensions examined in this study (e.g., IDV, PDI, UAI, and LTO). Specifically, this comparison illustrates how the key dimensions that were statistically associated with AI readiness in the main analysis—positive associations for IDV and LTO and negative associations for PDI and UAI—co-occur with high AI readiness in three high-scoring countries. The purpose is not to claim causality but to highlight patterns that are broadly consistent with the statistical associations between Hofstede’s dimensions and AI readiness. Accordingly, this comparison should be read as an interpretive complement to the quantitative analysis rather than evidence of causal mechanisms (see Table 3 for details).

**Table 3:** Comparison of AI readiness and cultural dimensions among high-scoring countries.

Country	AI readiness, rank	IDV: Individualism	LTO: Long-Term Orientation	PDI: Power Distance
United States	87.03, 1st	91 (very high)	26 (low)	40 (low)
Singapore	84.25, 2nd	20 (low)	72 (high)	74 (high)
Japan	75.75, 12th	46 (medium)	88 (very high)	54 (medium)

The US’s exceptionally high individualism is associated with a more decentralized, entrepreneurial ecosystem. In individualistic societies, personal freedom and achievement are highly valued, which translates into strong incentives—both social and economic—for innovation (Gorodnichenko and Roland, 2011). Empirical evidence shows that countries with high individualism have higher rates of innovation and opportunity-driven entrepreneurship (Assmann and Ehrl, 2021). This relationship is further supported by cross-national evidence that individualism is associated with higher national rates of innovation (Shane, 1993). In practice, this pattern is consistent with an AI ecosystem in the United States that features agile startups and autonomous R&D, where entrepreneurs freely experiment with new AI tools and business models and innovation is propelled by entrepreneurial experimentation and opportunity-driven entry. For example, awarding social status to innovators has been linked to faster growth (Gorodnichenko and Roland, 2011) and opportunity entrepreneurship (Assmann and Ehrl, 2021), reflecting the US’s vibrant startup culture.

This alignment is consistent with our finding that higher IDV and lower PDI are associated with higher AI readiness, suggesting that in such contexts, policies fostering bottom-up innovation may be particularly implementable.

In contrast, Singapore presents a markedly different model. Its cultural profile—low individualism, very high power distance, and high long-term orientation—aligns with a top-down, coordinated approach. In Hofstede’s framework, high-PDI societies accept strong hierarchical authority, while low-IDV cultures emphasize collective goals (Hofstede, 2025a,b). These cultural traits are consistent with strong public-sector leadership in national initiatives.

Reflecting this, Singapore’s government leads AI development with strategic incentives, institutional support, and regulation (Frana, 2024; Khanal et al., 2024). The official National AI Strategy emphasizes a key government role in curating incentives (e.g., targeted grants), resources (e.g., compute and talent), and regulatory frameworks across sectors (Singapore Government, 2023). This reflects a centralized, technocratic approach designed to mobilize stakeholders through coordinated planning rather than market spontaneity. Consistent with this, Singapore’s model also harnesses the productive energy of free-market capitalism contained within clear guardrails through stakeholder consensus-building and quasi-regulation (Allen et al., 2025).

Singapore’s high AI readiness coexists with systematic, state-led execution that is consistent with its high-PDI, high-LTO profile; such coordination may be particularly compatible with achieving and sustaining readiness in this cultural context.

Japan is often characterized as taking a relatively cautious stance toward AI, consistent with heightened concern about AI-related risks and a preference for careful evaluation of its impacts. In the Japan country results of a global AI trust survey, Japanese respondents report being more worried than optimistic about AI, and 70% report concern about negative outcomes from AI (Gillespie, 2025). Consistent with a broader risk-averse climate, individual-level evidence from Japan also shows that higher risk aversion is associated with a lower likelihood of engaging in angel investing, and prior research links risk aversion to reduced entrepreneurial interest and action (Honjo et al., 2024). Taken together, these patterns suggest a lower appetite for high-uncertainty, disruptive innovations and greater receptiveness to incremental technological advancement.

Nevertheless, such caution can coexist with meticulous and precise implementation; comparative innovation-management research reports that Japanese projects tend to rely on thorough planning and strict control mechanisms to minimize deviations during execution (Herstatt et al., 2006). In policy terms, Japan’s AI governance has emphasized voluntary, nonbinding guidance (“soft law”) and has prioritized international interoperability and alignment through frameworks such as the G7 and OECD (Ichikawa, 2025). More broadly, policy discussions often portray Japan’s approach as relatively incremental and coordination-oriented.

These cases collectively illustrate that high AI readiness is not contingent upon a single cultural configuration. Rather, distinct cultural profiles can support different, potentially equally effective models of AI adoption. Our findings indicate statistically significant associations between Hofstede’s cultural dimensions and national AI readiness. In this light, cultural traits are best treated as contextual parameters for policy design; aligning AI policy tools with prevailing cultural profiles may enhance implementability.

### 4.3 Limitations and future research

Several limitations should be acknowledged. First, this study relies on cross-sectional, correlational country-level data; accordingly, the observed relationships should be interpreted as associations rather than causal effects. Second, the analytic sample was restricted to 61 countries because only those countries were covered by both the Government AI Readiness Index and Hofstede’s cultural dimension dataset; this overlap may limit statistical power and may introduce selection bias if data availability is systematic across regions or income levels. Third, both the AI Readiness Index and Hofstede scores are secondary indicators with their own measurement assumptions, and Hofstede’s framework remains debated. Finally, because Hofstede’s dimensions can be intercorrelated, multicollinearity is a potential concern in cross-national models; however, in our sample, VIF/tolerance diagnostics did not suggest severe multicollinearity. Coefficients should nonetheless be interpreted cautiously given the small sample size and the use of secondary indicators.

Future research could further elucidate the mechanisms underlying these associations by combining cross-national models with structural covariates (e.g., GDP, digital infrastructure, education) and by applying approaches that address collinearity (e.g., PCA-derived factors or regularized regression). In addition, cross-cultural policy analyses comparing regulatory frameworks and public-sector capacities would be a valuable direction.

## Data Availability

Publicly available datasets were analyzed in this study. Is data can be found here: https://geerthofstede.com/research-and-vsm/dimension-data-matrix/.
