# Correction: The association between national culture and AI readiness: a cross-national study

**DOI:** 10.3389/frai.2026.1835185

**Published:** 2026-04-13

**Authors:** Frontiers Production Office

**Affiliations:** Frontiers Media SA, Lausanne, Switzerland

**Keywords:** artificial intelligence, cross-cultural studies, Hofstede's cultural dimensions, national culture, technology adoption

A correction has been made to the section **Introduction**, paragraph 3. The last sentence of the paragraph previously read: “The purpose of this paper is to systematically investigate this underexplored relationship (see Figure 1).”

The sentence now reads: “The purpose of this paper is to systematically investigate this underexplored relationship.”

The original version of this article has been updated.

